# Using Natural Dye Additives to Enhance the Energy Conversion Performance of a Cellulose Paper-Based Triboelectric Nanogenerator

**DOI:** 10.3390/polym16040476

**Published:** 2024-02-08

**Authors:** Supisara Piwbang, Walailak Kaeochana, Pawonpart Luechar, Weeraya Bunriw, Praphadsorn Chimsida, Wimonsiri Yamklang, Jirapan Sintusiri, Viyada Harnchana

**Affiliations:** 1 Department of Physics, Khon Kaen University, Khon Kaen 40002, Thailand; supisara.pi@kkumail.com (S.P.); walailakkaeochana@kkumail.com (W.K.); pawonpart_l@kkumail.com (P.L.); weeraya_b@kkumail.com (W.B.); praphadsorn_ch@kkumail.com (P.C.); wimonsiri_ya@kkumail.com (W.Y.); jsintusiri@kkumail.com (J.S.); 2 Institute of Nanomaterials Research and Innovation for Energy (IN-RIE), Khon Kaen University, Khon Kaen 40002, Thailand

**Keywords:** cellulose paper, natural dyes, triboelectric nanogenerator, energy harvesting

## Abstract

Green and sustainable power sources for next-generation electronics are being developed. A cellulose paper-based triboelectric nanogenerator (TENG) was fabricated to harness mechanical energy and convert it into electricity. This work proposes a novel approach to modify cellulose paper with natural dyes, including chlorophyll from spinach, anthocyanin from red cabbage, and curcumin from turmeric, to enhance the power output of a TENG. All the natural dyes are found to effectively improve the energy conversion performance of a cellulose paper-based TENG due to their photogenerated charges. The highest power density of 3.3 W/m^2^ is achieved from the cellulose paper-based TENG modified with chlorophyll, which is higher than those modified with anthocyanin and curcumin, respectively. The superior performance is attributed not only to the photosensitizer properties but also the molecular structure of the dye that promotes the electron-donating properties of cellulose.

## 1. Introduction

Paper-based electronics have gained increasing attention due to their many appealing aspects, including flexibility, light weight, degradability, eco-friendliness, recyclability, and low cost. Meanwhile, with advances in information technology and portable electronics, the demand for power supply for these devices is rising. The development of sustainable and renewable energy sources has become crucial. Regarding environmental issues, harvesting energy from the environment is one of the promising solutions. A triboelectric nanogenerator (TENG) is a newly developed technology for harnessing mechanical energy from ambient environments [1]. A TENG can convert waste mechanical energy into electricity with the working principle of a combination of contact electrification and electrostatic induction. A TENG offers numerous advantages including high energy conversion efficiency, multiple working modes, simple device fabrication, and low cost [2]. 

Paper is an important cellulose product extensively used in human everyday life. Cellulose is known as a versatile natural material, which can be extracted from plants and some species of bacteria and algae [3]. It is biodegradable, environmentally friendly, nontoxic, and low cost, making it the most desirable material for use in the paper industry. Besides the key advantages given above, cellulose papers have diversity for modification, a light weight, and good mechanical properties, rendering them an ideal material for fabricating a TENG. In recent years, great efforts have been made to explore cellulose-based TENGs for energy harvesting and sensing applications [4,5,6,7,8,9,10]. Cellulose paper has been recently reported for its highest record power density of over 300 W/m^2^ [11]. Based on the aforementioned benefits and its promising performance, cellulose paper-based TENGs show great potential for the development of the next generation power sources and paper-based electronic devices.

Since the electrical output of a TENG is governed by the triboelectric charge density [12], a number of approaches have been reported for boosting the power output of cellulose paper-based TENGs [13]. These include physical and chemical modifications [14], for example, constructing a hierarchical nanostructure to increase the surface area [15] and dielectric modulation with the addition of BaTiO_3_ [16], Fe_3_O_4_ [9], dielectric materials, and chemical functionalization [17,18]. Aside from the aforementioned approaches, triboelectric charge density is also enhanced through photogenerated charges under the illumination of light. The incorporation/hybrid of photoelectric materials with triboelectric materials, such as Si [19], ZnO [20], MoS_2_ [21], and MAPbl_3_ perovskite [22,23], were demonstrated to enhance the power output of TENGs through the photoinduced charges. 

Natural dyes are known for their photosensitizer properties [24] that can harvest sunlight and convert it into electricity. There are a number of natural dyes extracted from natural products such as plants, leaves, and fruits. In this regard, natural dyes have been widely explored as photosensitizers in dye-sensitized solar cells, replacing expensive synthetic photosensitizers [25,26,27], with the benefits of biodegradation, non-toxicity, environmental friendliness, and cost effectiveness. The most common natural dyes are chlorophyll [28], anthocyanin [29], carotenoid [30], and curcumin [31]. Considering a key role in the charge generation of natural dyes by converting light into electron–hole pairs, the incorporation of natural dyes is anticipated to promote the charge density of cellulose triboelectric materials. 

Sugarcane, the primary source for the sugar industry, is a major agricultural crop in many countries. Agricultural waste from sugarcane, especially its leaves, is typically removed by burning during the harvesting process before it is supplied to the sugar manufacturer. This common practice results in the generation of air pollution, notably contributing to the production of particulate matter 2.5 μm (PM2.5). Sugarcane leaves consist of approximately 40% cellulose, 25% hemicellulose, and 18–20% lignin [32,33]. Exploring the potential use of sugarcane waste is essential not only for addressing global environmental concerns but also for contributing to the principle of the bio-circular green (BCG) economy model. 

In this work, we propose a novel modification method for cellulose paper with natural dye additives to enhance the power output of TENGs. Natural dyes, including chlorophyll, anthocyanin, and curcumin, were extracted from spinach, red cabbage, and turmeric or curcuma longa, respectively. Cellulose papers derived from sugarcane leaves modified with natural dyes were employed as triboelectric layers for TENGs. The effects of the dye types and dye loading on TENG performance were investigated. The findings of our work could be useful for the development of high performance TENGs from natural-based materials, which shows potential for the development of power sources and next-generation paper-based electronic devices. 

## 2. Materials and Methods

### 2.1. Materials and Chemicals

Sugarcane leaves were obtained from a local sugarcane farm (Buriram, Thailand). Red cabbage, spinach leaves, and turmeric were purchased from a market (Khon Kaen, Thailand). Sodium hydroxide (NaOH, 97%) was purchased from KEMAUS (Cherrybrook, Australia). Hydrogen peroxide (H_2_O_2_, 35%) was purchased from ANaPURE (Brightchem Sdn. Bhd., Rawang, Selangor, Malaysia).

### 2.2. Preparing Cellulose Fibers 

Cellulose fibers were extracted from sugarcane leaves, as described in our previous work [9]. Briefly, sugarcane leaves were firstly washed and dried in an oven at 60 °C for 48 h. The dried sugarcane leaves were then cut and ground with a grinder machine to obtain powders with an approximate size of 200 µm. The ground powers underwent alkaline treatment by mixing 30.0 g of leaf powder with 10% NaOH at a solid/liquid ratio of 1:20 g/mL. The mixture was then heated at 90 °C for 4 h. After that, the mixture was filtered and washed with DI water until a neutral pH was obtained. The alkaline-treated product then underwent bleaching treatment, using alkaline peroxide as the bleaching agent, which was prepared from a mixture of 25% H_2_O_2_ and 2% NaOH solutions (at a H_2_O_2_ to NaOH volume ratio of 4:1). The bleaching step was performed at 90 °C for 5 h. The product was then washed with DI water to reach a neutral pH, and the slurry of CF microfibers was obtained.

### 2.3. Natural Dye Extraction 

Natural dyes were extracted from three different natural products including spinach, red cabbage, and turmeric, as follows. All natural products were washed and ground with a grinder machine. The extraction of dyes from spinach and turmeric was performed by using ethanol as a solvent, with DI water for red cabbage. All the natural dyes were prepared in three different concentrations by varying the solid-to-liquid ratios (S:L in g/mL) from 1:1, 2:1, to 3:1. The mixture of ground natural product and solvent was sonicated for 30 min and then filtered to obtain the natural dye, which was subsequently used as an additive for cellulose as described below.

### 2.4. Preparation of Cellulose Papers with the Incorporation of Natural Dyes

First, 3 g of cellulose slurry was mixed with 1 mL of the prepared natural dye solution described in Section 2.3. The mixture was then stirred with a stainless steel spatula to obtain a uniform suspension. The paste was then cast on a 4 × 4 cm^2^ ITO substrate to achieve a thickness of approximately 1 mm. Three specimens were prepared for each experimental condition. The specimens were then dried at 60 °C for 12 h; then, the samples were ready for TENG performance testing. The cellulose paper (CP) with the additions of natural dyes were labelled as CP@Spinach, CP@Red cabbage, and CP@Turmeric followed by the concentrations at an S:L of 1:1, 2:1, and 3:1.

### 2.5. Characterizations

The morphologies and crystal structure were investigated using a field emission scanning electron microscope (FE-SEM) (Helios Nanolab G3 CX, FEI, Crawley, Australia). Chemical structure analysis was performed using Fourier-transform infrared spectroscopy (FTIR) (TENSOR27, Markham, ON, Canada). The UV–vis absorption spectra were probed using a UV–vis spectrometer (UV-1800, Shimadzu, Kyoto, Japan).

### 2.6. TENG Output Measurement

The prepared cellulose papers on ITO substrates were used as tribopositve materials, and a Teflon sheet was used as a contact tribonegative material with the contact areas of 4 × 4 cm^2^. The electrical outputs were acquired with a single electrode operation mode under an impact force of 4 N, at a frequency of 5 Hz. The output voltage and current were measured using an oscilloscope (Tektronix DPO2002B, Tektronix China Ltd., Shanghai, China) and a digital ammeter (Keithley DMM6500, Tektronix China Ltd., Shanghai, China), respectively.

## 3. Results

**Figure 1:**
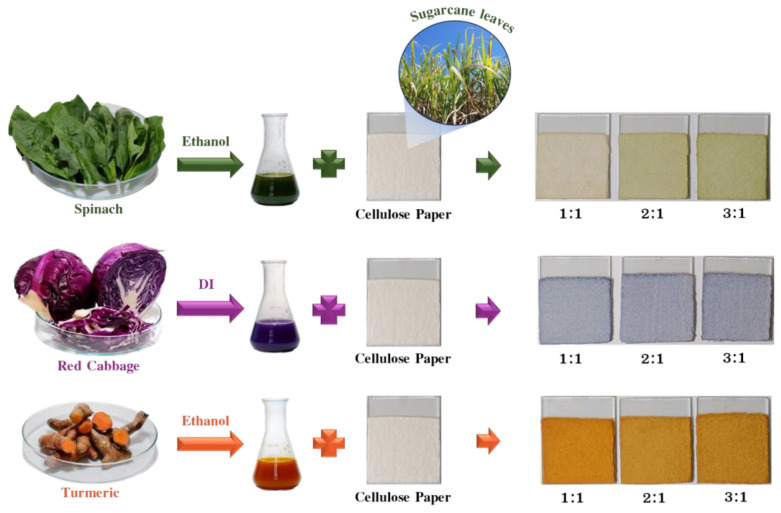
Summary of the cellulose paper modification process using natural dyes extracted from spinach, red cabbage, and turmeric.

The fabrication process for cellulose papers modified with natural dyes is summarized in Figure 1. Natural dyes were extracted from spinach, red cabbage, and turmeric. Dye solutions were prepared with varying S:L ratios of 1:1, 2:1, and 3:1 to achieve different dye concentrations. These extracted dyes were then added to cellulose fibers derived from sugarcane leaves and cast onto ITO glass substrates for TENG performance testing. The resulting papers exhibited colors corresponding to the extracted dyes: green for the CP@Spinach, violet for the CP@Red cabbage, and orange for the CP@Turmeric. The paper intensity of paper colors increased with higher dye concentrations. 

**Figure 2:**
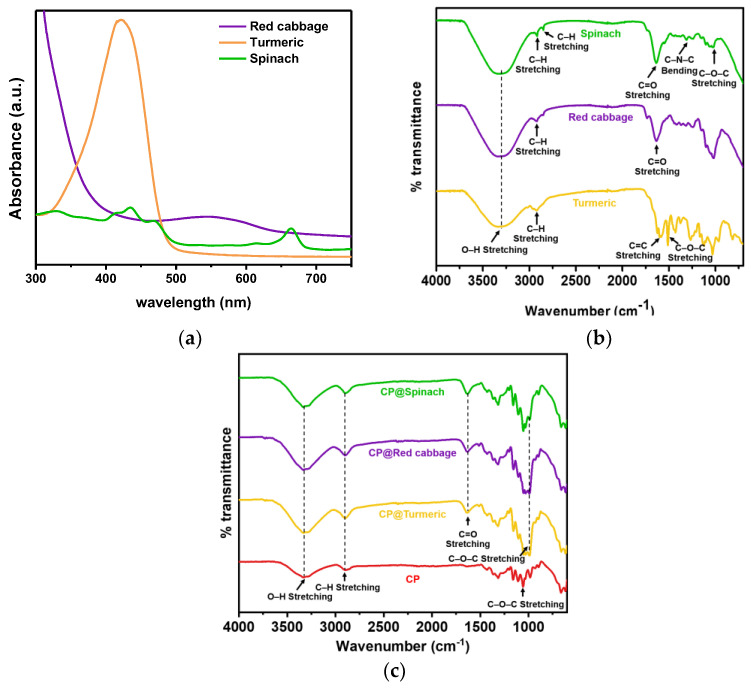
(**a**) UV–vis absorption spectra and (**b**) FTIR transmittance spectra of the extracted dyes from spinach, red cabbage, and turmeric. (**c**) FTIR transmittance of cellulose papers modified with natural dyes extracted from spinach (CP@Spinach), red cabbage (CP@Red cabbage), and turmeric (CP@Turmeric).

The extract dyes were characterized by UV–vis absorption spectroscopy and FTIR spectroscopy, as illustrated in Figure 2a and Figure 2b, respectively. All the natural dyes showed absorption peaks in the visible light region. The absorption spectrum of the spinach extract dye exhibited multiple absorptions, including a band at 410–460 nm and a peak at 663 nm, corresponding to the absorptions of chlorophyll a and chlorophyll b [34]. The absorption spectrum of the turmeric dye showed a broad band with a maximum absorbance peak at 421 nm, associated with the π-π* excitation of curcumin [31,35]. The red cabbage dye exhibited a broad absorption band from 450 to 650 nm, with absorption maxima at 550 nm, assigned to the absorption of anthocyanin [36]. 

The FTIR analysis of the extracted dyes from spinach, red cabbage, and turmeric is displayed in Figure 2b. All the natural dyes showed the presence of hydroxyl groups, as evidenced by the observed O–H stretching at 3318 cm^−1^. The chlorophyll dye from spinach showed CH_3_ vibration at 2915 cm^−1^, CH_2_ vibration at 2848 cm^−1^, C=O vibration at 1637 cm^−1^, C–O–C vibration at 1022 cm^−1^, and C–N–C bending at 1312 cm^−1^ [37,38]. Anthocyanin from red cabbage exhibited vibrations from C–H stretching at 2917 cm^−1^, C=O stretching at 1637 cm^−1^, and C–O–C stretching at 1022 cm^−1^ [38,39]. Curcumin from turmeric displayed the vibrations from CH_3_ stretching at 2915 cm^−1^, C=C stretching at 1589 cm^−1^, and C–O–C at 1508 and 1029 cm^−1^ [40,41]. The chemical structures of the dye molecules with the observed functional groups and their corresponding wavenumbers are displayed in Table 1. 

The chemical structures of the cellulose papers after modification with natural dyes are presented in Figure 2c. The FTIR spectrum of cellulose paper exhibited prominent absorption at 3330, 2896, 1309, and 1056 cm^−1^, associated with the vibration from O–H, C–H, C–H wagging, and C–O–C stretching, respectively [42]. All the modified cellulose papers with natural dyes showed pronounced absorption peaks of O–H stretching at 3330 cm^−1^, C=O stretching at 1637 cm^−1^, and C–O–C stretching at 1053 and 993 cm^−1^, attributed to the presence of natural dyes, consistent with their FTIR absorptions (Figure 2b). 

**Figure 3:**
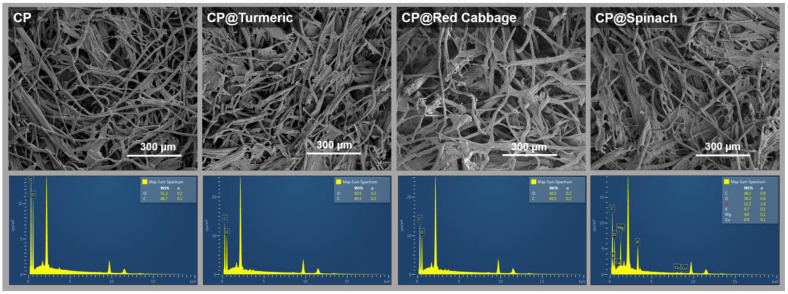
SEM images and EDX spectra with elemental analysis of the cellulose paper (CP) and the cellulose paper modified with natural dyes extracted from spinach (CP@Spinach), red cabbage (CP@Red Cabbage), and turmeric (CP@Turmaric).

The SEM images showing the EDS elemental analysis of cellulose papers modified with natural dyes are presented in Figure 3. Fiber structures were observed in all specimens, and EDX elemental analysis confirmed the presence of carbon (C) and oxygen (O) characteristic of cellulose material. Sheet structures were also observed in the cellulose papers modified with natural dyes, suggesting the incorporation of dye-extracted materials. The EDX elemental analysis of all the modified cellulose papers revealed a relatively similar elemental composition, with carbon and oxygen being the main constituents. Additionally, traces of nitrogen (N) and magnesium (Mg) were detected in the CP@Spinach specimen, attributed to the presence of chlorophyll molecules. 

**Figure 4:**
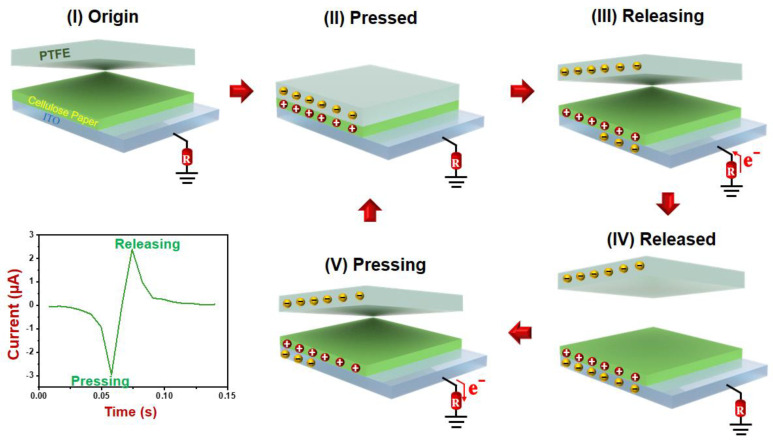
Schematic illustration of the generation of electricity of a TENG operated in a single electrode mode.

The evaluation of the energy conversion performance was performed by measuring the electrical output voltage and current of the fabricated TENG. The operation of the cellulose paper TENG in a single electrode mode is illustrated in the schematic shown in Figure 4. The cellulose paper served as the triboelectric layer coated on the ITO conductive electrode and was tested with a PTFE sheet as the pair triboelectric material. The electricity generation of the TENG relies on a combination of two physical phenomena known as contact electrification and electrostatic induction, which is described as follows: In state I, when the PTFE sheet is pressed into contact with the cellulose paper’s surface, electrons are transferred from the cellulose paper to the PTFE due to the high electron affinity of PTFE. This electrification effect results in the formation of a negative surface charge on the PTFE and a positive charge on the cellulose paper’s surface. The separation of the two surfaces creates a potential difference, leading to electrostatic induction. Electrons flow from the ground to balance the created potential, generating the positive electrical current. When the two surfaces come into contact again, the potential difference is reduced, and electrons flow back to the ground, creating a negative current signal. The generated electrical current is depicted in the inset of Figure 4. 

**Figure 5:**
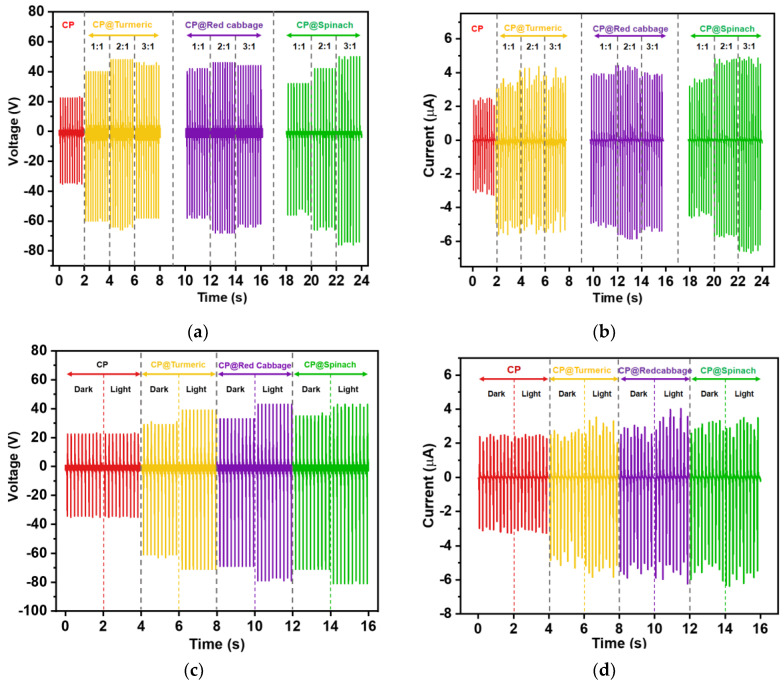
(**a**) Output voltage and (**b**) output current of CP TENG and CP@Turmeric, CP@Red cabbage, and CP@Spinach TENGs with different dye concentrations prepared at S:L ratios of 1:1, 2:1, and 3:1. (**c**) Output voltage and (**d**) output current of the CP TENG and CP@Turmeric, CP@Red cabbage, and CP@Spinach TENGs when tested in dark and light condition.

The modified cellulose papers showed a significant improvement in the TENG electrical outputs, as presented in Figure 5a,b, which represent the output voltage and current, respectively. The peak-to-peak output voltage and current of all the cellulose paper TENGs are listed in Table 2. This enhancement in TENG outputs was attributed to the incorporation of natural dyes, light-absorbing molecules, which are able to convert sunlight into electricity. The excited electrons generated from the dye molecules when absorbing light contributed to the enhancement in the triboelectric charges in the cellulose papers. Upon comparing the highest electrical outputs of the TENG modified with three different types of natural dyes, chlorophyll was found to be more effective in enhancing the electrical output of the CP TENG than the other two dyes. The CP@Spinach 3:1 TENG achieved the highest electrical outputs of 126 V and 11.4 µA, surpassing those of the unmodified CP TENG (57 V and 5.7 µA). 

Note that the S:L ratios may not be directly proportional to the concentration of the dye pigments; consequently, the TENG performance cannot be directly compared between the cellulose papers modified with different dye additives. Considering CP@Tumeric and CP@Red cabbage TENGs, the optimum TENGs were CP@Tumeric 2:1 and CP@Red 2:1. The higher dye loading (S:L) in the CP@Tumeric 3:1 and the CP@ Red cabbage 3:1 adversely affected the TENG performance. It is possible that a high dye content could affect the morphology of the cellulose surface, as evidenced by the SEM images (Figure 3), where the dye extract pulps filled the fiber structure of the cellulose. However, this was not the case for the CP filled with chlorophyll. The higher the chlorophyll loading, the higher the TENG electrical output. 

According to the molecular structure of chlorophyll, four nitrogen atoms coordinate with the central Mg atoms, acting as electron donors [43]. The presence of a high chlorophyll content not only promotes the conversion of photons into charge carriers but also intensifies the positive triboelectric charges owing to the electron donor ability of the chlorophyll molecule. The increased triboelectric charges contribute to the enhanced TENG electrical output, as described by Equations (1) and (2). The open circuit voltage (*V_oc_*) and short circuit current (*I_sc_*) depend on the triboelectric charge density (*σ*), the size of the contact area (*S*), and the effective thickness constant (*d*_0_), as well as the distance between the friction surfaces (*x*(*t*)) [44].

(1) $$V_{oc}=\frac{\sigma x\left(t\right)}{\varepsilon_{0}}$$

(2) $$I_{sc}=\frac{\sigma Sd_{0}}{{\left(d_{0}+x\left(t\right)\right)}^{2}}\frac{dx\left(t\right)}{dt}$$

The main contribution to the electrical output of the CP TENG in this work was the triboelectric charge density, which was enhanced by the photogenerated charges initiated by the incorporation of natural dyes as discussed above. 

The effect of the photo-enhanced charge generation on the TENG performance was studied by performing the electrical output measurement on the CP, CP@Turmeric 2:1, CP@Red cabbage 2:1, and CP@Spinach 3:1 TENGs in both light and dark conditions. The electrical output voltage and current of the CP and natural dye-modified CP TENGs are presented in Figure 5c,d. The TENG performance of the unmodified CP TENG remained consistent when measured in both dark and light conditions, while all the modified CP TENGs showed a significant improvement with the irradiation of light. This indicated that the natural dyes played a significant role in promoting electrical charges due to their photosensitizing properties. Under the irradiation of light, the CP@Turmeric 2:1, CP@Red cabbage 2:1, and CP@Spinach 3:1 TENGs demonstrated output enhancements of 1.22, 1.19, and 1.17 times, respectively, compared to their performance in dark conditions. This implies that the performance enhancement contributed by the photosensitizing properties of the three different dyes were comparable. It is suggested that the superior performance of the CP@Spinach is not only attributed to the photogenerated charges but also the electron donor ability of the chlorophyll molecule, as discussed above.

Typically, the change in the output current is proportional to that of the output voltage. However, it was observed that the changes in the output current were smaller than those in output voltage when exposed to light in the modified CP TENG. This discrepancy may be attributed to the poor adhesion between the CP specimens and the ITO conductive glass, which adversely affected the electrostatic induction, resulting in a small change in the output current. 

**Figure 6:**
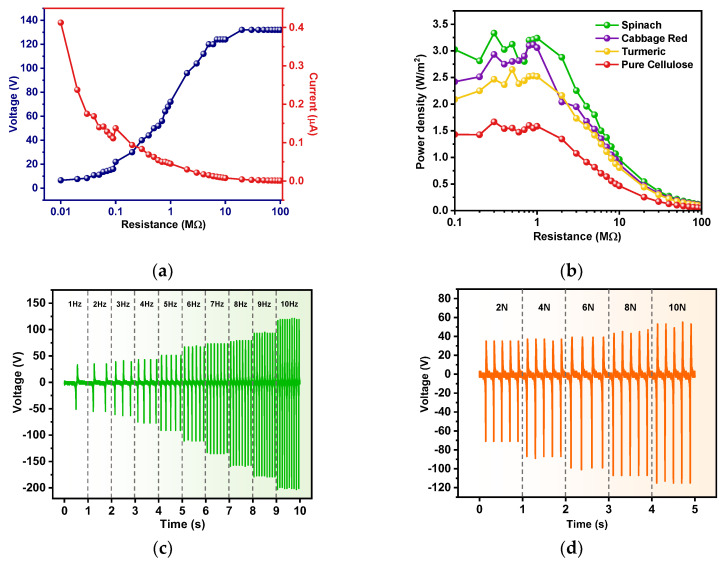
(**a**) Plot of the output voltage and current and (**b**) the corresponding power density of CP@Spinach TENG at various external load resistances. (**c**) Frequency and (**d**) impact force dependence of the CP@Spinach TENG performance.

The power density, which describes the electrical power delivered per unit area of TENG, was examined. The plot of the output voltage and current versus the load resistances of the CP@spinach (3:1) TENG is presented in Figure 6a, indicating an increase in the voltage output and a decrease in the current output with increasing load resistances, in accordance with Ohm’s law. The CP@spinach TENG exhibited the highest power density of 3.33 W/m^2^ at a matched load of 0.3 MΩ, surpassing the values for CP@tumeric and CP@red cabbage TENGs, which were 2.65 and 3.10 W/m^2^, respectively, as shown in Figure 6b and Table 3. The power densities of the modified CP TENGs were approximately two times greater than that of the unmodified CP TENG.

The working frequency and force dependence of the electrical outputs of the CP@Spinach TENG were also studied. The plot of the electrical output voltage as a function of the impact frequency and force is presented in Figure 6c,d. As the impact frequency increased from 1 to 10 Hz, while the impact force was kept constant at 5 N, the electrical outputs increased from 84 V and 7.9 µA at 1 Hz to 324 V and 25 µA at 10 Hz, as shown in Table S1 in the Supplementary Material. This suggests that the TENG was able to effectively generate electricity at relatively low operation frequencies. The enhanced electrical output of the TENG with increasing working frequency was attributed to the accumulation of the surface charge due to a shorter contact–separation cycle at higher frequencies [45]. The TENG electrical output exhibited an increasing trend with an applied impact force, tested from 2 to 10 N. The measured outputs increased from 106 V and 8.6 µA at an impact force of 2 N to 168 V and 14 µA at 10 N, as shown in Table S2 in the Supplementary Material. This increment was accounted for by the deformation and the enhanced surface contact area under compression. 

**Figure 7:**
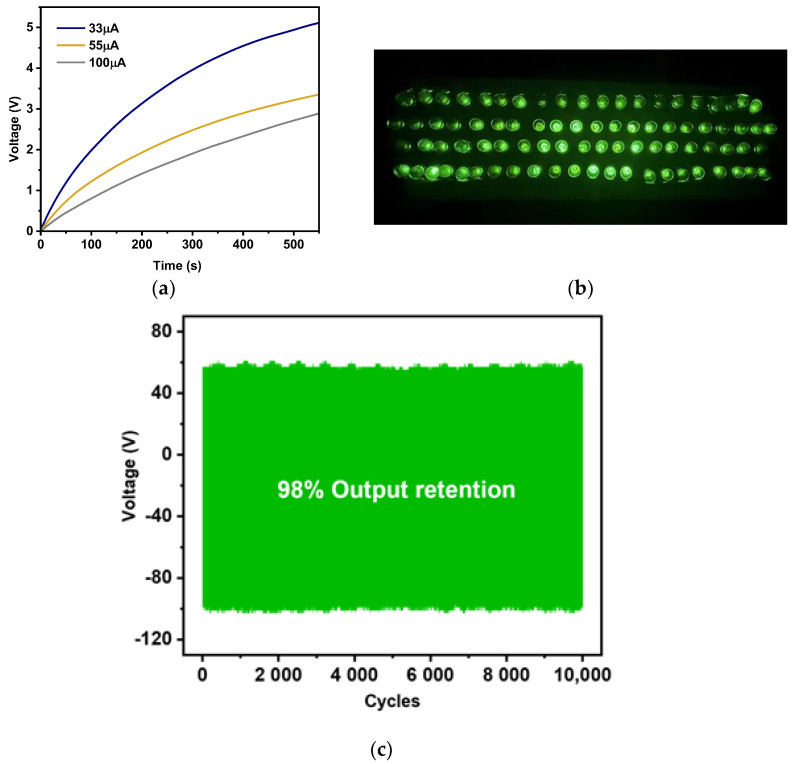
(**a**) Voltage profile of commercial capacitors when charged by the CP@Spinach TENG. (**b**) Digital photograph of 80 green LEDs illuminated by the generated electricity from the fabricated paper TENG. (**c**) Electrical output voltage tested under a constant impact force of 4 N over 10,000 cycles.

The application of TENG as a power source was demonstrated by charging a commercial capacitor via a bridge rectify circuit. The plot of the charged voltage versus time for commercial capacitors with three different capacitances, 33, 55, and 100 µF, is presented in Figure 7a. The larger the capacitor was, the longer the charging time. To attain 1.5 V, the capacitors of 33, 55, and 100 µF required charging times of 70, 140, and 200 s, respectively. The generated electrical output was able to instantaneously light up 80 green LEDs, as presented in Figure 7b. The output stability was also tested by applying an impact force of 4 N at a frequency of 5 Hz. The measured electrical output voltage over 10,000 cycles showed impressive stability, with a performance retention of 98%, as shown in Figure 7c. This suggests that the fabricated cellulose paper TENG exhibits good robustness, making it suitable for practical applications.

## 4. Conclusions

Natural-based TENGs were developed from cellulose paper fabricated from waste sugarcane leaves. The cellulose papers were modified with three different types of natural dyes extracted from spinach, red cabbage, and turmeric. The extracted dyes contained chlorophyll, anthocyanin, and curcumin. These natural dyes were found to promote the electrical output of the cellulose paper TENG due to their photosensitizing properties, which convert light into charge carriers, contributing to the enhanced triboelectric charge density. The highest power density of 3.3 W/m^2^ was obtained from the TENG fabricated from cellulose paper modified with chlorophyll extracted from spinach. This enhancement was attributed not only to the photogenerated charges but also to the electron-donating property of the chlorophyll molecules. 

## Figures and Tables

**Table 1:** Chemical structures, FTIR absorption wavenumber, and the corresponding functional groups of chlorophyll, anthocyanin, and curcumin.

Natural Dyes	Chemical Structures	Wavenumber (cm^−1^)	Functional Group
Chlorophyll	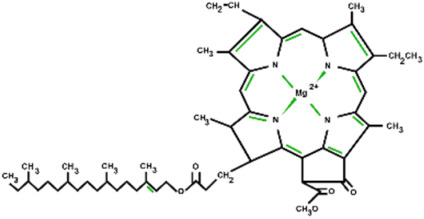	3318	O-H stretching
		2915	C-H stretching
		2848	C-H stretching
		1637	C=O stretching
		1312	C-N-C bending
		1022	C-O-C stretching
Anthocyanin	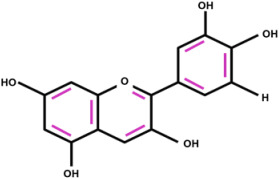	3318	O-H stretching
		2917	C–H stretching
		1637	C=O stretching
		1022	C-O-C stretching
Curcumin	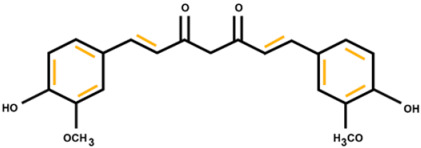	3318	O-H stretching
		2915	C-H stretching
		1589	C=C stretching
		1508	C-O-C stretching
		1029	C-O-C stretching

**Table 2:** Electrical output voltage (*V_pp_*) and current (*I_pp_*) of the CP, CP@Turmeric, CP@Red cabbage, and CP@Spinach TENGs at different S:Ls.

TENGs	S:L	*V_pp_* (V)	*I_pp_* (µA)
CP	-	57	5.7
CP@Turmeric	1:1	100	8.8
	2:1	114	9.6
	3:1	102	9.2
CP@Red cabbage	1:1	100	8.9
	2:1	114	10.2
	3:1	108	9.4
CP@Spinach	1:1	88	8.0
	2:1	108	10.5
	3:1	126	11.4

**Table 3:** The maximum power density of CP, CP@Turmeric, CP@Red cabbage, and CP@Spinach TENGs with the corresponding matched loads.

TENGs	Power Density (W/m^2^)	Matched Load (MΩ)
CP	1.9	0.01
CP@Turmeric	2.6	0.5
CP@Red cabbage	3.1	0.9
CP@Spinach	3.3	0.3

## Data Availability

Data are contained within the article and Supplementary Materials.

## Supplementary Materials

The following supporting information can be downloaded at: https://www.mdpi.com/article/10.3390/polym16040476/s1, Table S1: TENG outputs at different working frequencies; Table S2: TENG outputs at different impact forces.
