# Supplementary material for: Using Natural Dye Additives to Enhance the Energy Conversion Performance of a Cellulose Paper-Based Triboelectric Nanogenerator
Source: Polymers (Basel). 2024 Feb 8;16(4):476. doi: 10.3390/polym16040476 (PMC10892896; doi:10.3390/polym16040476)
Supplement: Supplementary file 1 [file polymers-16-00476-s001.zip › polymers-2831163-supplementary.pdf]

Supplementary materials

Using Natural Dye Additives to Enhance the Energy Conversion Performance of a Cellulose Paper-Based Triboelectric Nanogenerator

Supisara Piwbang, Walailak Kaeochana, Pawonpart Luechar, Weeraya Bunriw, Praphadsorn Chimsida, Wimonsiri Yamklang, Jirapan Sintusiri and Viyada Harnchana

Table S1. Output voltage and current of the CP@Spinach TENG at working frequecy of 1-10 Hz.

Frequency (Hz)	V_{pp} (V)	I_{pp} (μA)
1	84	7.9
2	90	8.6
3	104	9.6
4	120	12
5	142	16
6	178	17
7	208	18
8	238	19
9	270	22
10	324	25

Table S2. Output voltage and current of the CP@Spinach TENG at various impact forces ranging from 2-10 N.

Impact force (N)	V_{pp} (V)	I_{pp} (μA)
2	106	8.6
4	126	10
6	140	11
8	150	12
10	168	14
